# Tight Junction Proteins and the Biology of Hepatobiliary Disease

**DOI:** 10.3390/ijms21030825

**Published:** 2020-01-28

**Authors:** Natascha Roehlen, Armando Andres Roca Suarez, Houssein El Saghire, Antonio Saviano, Catherine Schuster, Joachim Lupberger, Thomas F. Baumert

**Affiliations:** 1 Institut de Recherche sur les Maladies Virales et Hépatiques, Inserm UMR1110, F-67000 Strasbourg, France; natascha.roehlen@etu.unistra.fr (N.R.); andres.roca-suarez@etu.unistra.fr (A.A.R.S.); elsaghire@unistra.fr (H.E.S.); saviano@unistra.fr (A.S.); catherine.schuster@unistra.fr (C.S.); joachim.lupberger@unistra.fr (J.L.); 2 Université de Strasbourg, F-67000 Strasbourg, France; 3 Pôle Hepato-digestif, Institut Hopitalo-universitaire, Hôpitaux Universitaires de Strasbourg, F-67000 Strasbourg, France

**Keywords:** Claudin, occludin, blood-biliary barrier, chronic liver disease, hepatocellular carcinoma, cholangiocellular carcinoma, NISCH syndrome

## Abstract

Tight junctions (TJ) are intercellular adhesion complexes on epithelial cells and composed of integral membrane proteins as well as cytosolic adaptor proteins. Tight junction proteins have been recognized to play a key role in health and disease. In the liver, TJ proteins have several functions: they contribute as gatekeepers for paracellular diffusion between adherent hepatocytes or cholangiocytes to shape the blood-biliary barrier (BBIB) and maintain tissue homeostasis. At non-junctional localizations, TJ proteins are involved in key regulatory cell functions such as differentiation, proliferation, and migration by recruiting signaling proteins in response to extracellular stimuli. Moreover, TJ proteins are hepatocyte entry factors for the hepatitis C virus (HCV)—a major cause of liver disease and cancer worldwide. Perturbation of TJ protein expression has been reported in chronic HCV infection, cholestatic liver diseases as well as hepatobiliary carcinoma. Here we review the physiological function of TJ proteins in the liver and their implications in hepatobiliary diseases.

## 1. Introduction

Tight junctions (TJ) are protein complexes on epithelial cells in all organs of the body and establish paracellular diffusion barriers between different compartments. The distinct cell polarity and selective paracellular diffusion hereby provides the molecular basis of tissue homeostasis [1]. Structurally, TJs consist of transmembrane proteins that function as the diffusion barriers and cytosolic proteins that interface the junctional complexes with the cytoskeleton [1]. While initially TJs were believed to serve as simple paracellular gates, in the past years, accumulating data have identified additional functions of TJs proteins. By maintaining cellular differentiation, intercellular communication as well as assembly of signaling proteins, TJ proteins have been shown to orchestrate inside-out and outside-in signaling, hereby affecting cell proliferation, migration, apoptosis, and inflammation [2,3,4]. On the other hand, several growth factors, cytokines, and signaling cascades induce and regulate localization and expression of TJ proteins, hereby affecting epithelial differentiation and barrier integrity [5,6].

In the healthy liver, TJ proteins are expressed on hepatocytes, cholangiocytes, and nonparenchymal cells such as endothelial cells [5,7,8]. While TJ proteins on hepatocytes build the blood-biliary barrier (BBIB) and are hijacked during hepatitis C virus (HCV) infection, TJ proteins on cholangiocytes line the intrahepatic bile ducts [7,9,10]. Besides their localization at the apical membrane, TJ proteins have also been described to be localized at the basolateral membrane and in the cytoplasm of hepatocytes. In these non-junctional localizations, TJ proteins regulate cell-matrix interactions, intracellular signaling and proliferation, migration, and invasion [11]. Perturbation of TJ structure, protein expression, and localization have frequently been described in chronic liver and biliary diseases, indicating their fundamental role in liver biology [12]. This review provides an overview of TJ proteins being expressed in the liver, their function in maintaining TJ structure and cell signaling outside of TJs, as well as their implication in hepatobiliary diseases.

## 2. Biology of Tight Junction Proteins

### 2.1. Structure and Composition of Tight Junctions

**Figure 1:**
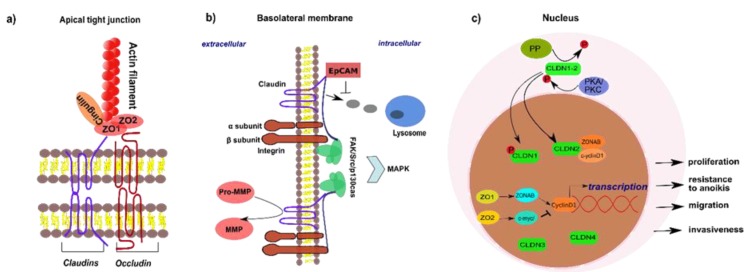
Functions of tight junction proteins at different subcellular localizations. Tight junction proteins are expressed at three different locations within epithelial cells with different functions including the apical membrane (**a**), the basolateral membrane (**b**), and in the nucleus (**c**). (**a**) At the apical membrane, tight junctions (TJs) are typically built by integral membrane proteins of the CLDN or Marvel-domain containing protein family (e.g., occludin—OCLN) that connect via C-terminus bound adapter proteins to intracellular actin filaments. (**b**) In the normal intestinal mucosa and in various cancer cell types, basolateral localized CLDNs have been found to regulate activation of pro-MMPs into MMPs and to interact with integrins at focal adhesion complexes, hereby affecting main intracellular signaling cascades such as the MAPK pathway. Investigations on colon cancer cell lines indicate EpCAM to specifically stabilize expression of CLDN1 and 7 at the basolateral membrane and to prevent their lysosomal degradation. (**c**) Nuclear localization has been reported for ZO1 and ZO2 as well as CLDN1-4 in various cancer cell types and is regulated by posttranslational modification. Within the nucleus, CLDN2 retains cyclinD1 and ZONAB hereby enhancing cell proliferation. Specific interaction of ZO1 with the transcription factor ZONAB regulates G1/S-phase progression by increasing cyclin D1, while ZO2 inhibits transcription of cyclin D1 by binding to c-myc. CLDN (Claudin); c-myc (MYC proto-oncogene); EpCAM (epithelial cell adhesion molecule); FAK (focal adhesion kinase); MAPK (Mitogen-activated protein kinase); MMP (Matrix-metalloproteinase); PKA (protein kinase A); PKC (protein kinase C); PP (protein phosphatase); Src (steroid receptor coactivator); ZO1 (Zonula occludens 1); ZO2 (Zonula occludens 2); ZONAB (ZO1-associated nucleic acid binding protein).

Tight junctions are shaped by intercellular protein-protein complexes connecting plasma membranes of neighboring cells. Thus, TJs often appear as ‘‘kissing points’’ by electron microscopy. Two models of TJ structure exist: the protein model and the protein-lipid hybrid model. The protein model postulates construction of the junctional diffusion barrier by transmembrane proteins on both sides, interacting in a homotypic or heterotypic way (shown in Figure 1a), whereas the hybrid model proposes membrane hemifusions built by inverted lipid micelles and stabilized by transmembrane proteins [1]. Yet no consensus on the ultrastructural appearance has been reached. However, in both cases, TJs build a regulatory semipermeable gate that enables selective paracellular diffusion depending on the size and charge of the corresponding molecule [1]. Moreover, TJs form an intramembrane barrier (also referred to as ‘‘fence function’’), that restricts exchange between the cells’ apical and basolateral surfaces [13]. However, whether the fence function of TJs is critical or not for the establishment of a polarized phenotype has been a matter of debate, taking into account that it has been observed how epithelial cells are able to polarize in the absence of cell-cell junctions [14,15].

The transmembrane domains of TJs on epithelial cells are mainly built by tetraspanin-associated proteins of the claudin (CLDN) family and the junctional proteins occludin (OCLN) and MarvelD3, which contain a MAL and related proteins for vesicle trafficking and membrane link (MARVEL) domain. Moreover, junctional adhesion molecules (JAMs) have been reported as integral membrane proteins in TJs [16,17]. Tricellular TJ proteins characterize cell adhesion between three neighboring cells and include tricellulin [18], lipolysis-stimulated lipoprotein receptor (LSR) [19], as well as immunoglobulin-like domain containing receptor (ILDR1 and ILDR2) [20]. Representatives of the cytosolic junctional plaque on the other hand are adapter proteins as Zonula occludens 1-3 (ZO1-3), membrane-associated guanylate kinase inverted (MAGI) proteins, and cingulin [1] (Figure 1a).

OCLN was the first identified transmembrane protein in TJs and belongs to the large protein family of Marvel-domain-containing proteins [21]. In contrast to the multiple and differentially expressed members of CLDN family, only one OCLN transcript has been described, which however occurs in differently spliced variants. With a size of 65 kDa, OCLN contains four transmembrane domains, one small intracellular loop, two extracellular loops, and intracellular localized C and N terminals (Figure 1a) [22].

The family of CLDN proteins comprises 27 members in mammals [23]. According to their physiological role in paracellular permeability, CLDNs can further be subgrouped into sealing CLDNs (CLDN1, 3, 5, 11, 14, and 19), cation-selective (CLDN2, 10b and 15) and anion-selective paracellular channel forming CLDNs (CLDN10a and 17), as well as water-permeable CLDNs (CLDN2 and 15). For the remaining CLDNs, their roles on epithelial barriers are not yet fully understood [24]. These 20–27 kDa proteins consist of four transmembrane domains, two extracellular loops, and a cytoplasmatic carboxyl tail (Figure 1a). As integral proteins of TJs, CLDNs are reported to regulate ion and water permeability of the paracellular barrier [1,25,26]. 

With four transmembrane domains, cytoplasmatic C- and N-terminals, and two extracellular loops, tricellulin shows strong structural similarity to CLDNs and OCLN [18,27]. While OCLN and CLDN represent the main transmembrane proteins of apical cell adhesions between two cells (bicellular tight junction, bTJ), tricellulin is mainly enriched at tricellular contact regions (tricellular tight junction, tTJ), although also been identified in bTJs [18]. LSR, ILDR1 and 2, which are commonly described as the angulin family, have been reported to recruit tricellulin to tTJ [20].

JAMs belong to the immunoglobulin superfamily (IgSF). Originally discovered on leucocytes as key players of leucocyte-endothelial cell interaction and trans-endothelial migration, JAM-A-C as well as the related IgSF members CAR, endothelial cell-selective adhesion molecule (ESAM), and JAM-4 were later described to be enriched in epithelial and endothelial TJs. Consisting of two IgSF domains, two Ig-like domains, one single transmembrane domain, and a PDZ-domain binding cytoplasmatic tail, these proteins contribute to barrier formation and TJ associated signaling [16,17].

Besides transmembrane proteins, TJs consist of junctional plaque components that connect the junctional membrane with the cytoskeleton. ZO proteins are the most important adapter proteins, that connect CLDN, OCLN, and tricellulin with the cytoskeleton, hereby enabling clustering of protein complexes to the intracellular domains of TJs (Figure 1a). Apart from TJs, ZO proteins have also been described in cadherin-based adherens junctions and gap junctions [28]. Three ZO proteins (ZO1-3) with high structural similarity have been discovered. ZO1, the best described member of the family of ZO proteins represents a 220 kDa scaffolding protein, that includes three types of functional domains, a Src homology 3 domain (SH3), three PDZ domains, a proline rich and a guanylate kinase domain [29,30]. ZO proteins directly interact with the intracellular actin filaments and the first PDZ domain has been shown to associate with the C-terminus of CLDN and OCLN proteins, hereby regulating TJ assembly (Figure 1a) [31,32]. Other representatives of the junctional plaque are cingulin and 7H6 [33,34]. For a detailed review regarding the general structure and composition of TJs see [35,36].

The TJ complex is known to be highly dynamic with continuous remodeling by clathrin-mediated endocytic recycling [37,38,39,40]. Recycled or newly produced TJ proteins are sorted in the Golgi-network and transported by specific trafficking proteins to the desired localizations [41,42]. On the other hand, several growth factors, cytokines, and signaling cascades induce and regulate localization and expression of TJ proteins, hereby affecting epithelial differentiation and barrier integrity [5,6].

Knockout (KO) studies in cultured epithelial cells indicate an increase of paracellular permeability by loss of single CLDN proteins [43,44]. In contrast, KO of OCLN does not alter baseline barrier function, but attenuates cytokine-induced increase in trans-epithelial resistance [45]. Knockdown of tricellulin using siRNA decreases trans-epithelial electrical resistance and increases the paracellular permeability in cultured epithelial cells [18]. JAM-A in vitro and in vivo KO studies revealed increased epithelial permeability potentially due to perturbed regulation of CLDN expression and induction of apoptosis [46,47]. Loss of ZO1 retards but not completely hampers TJ formation, probably due to compensatory upregulation of ZO2. Thus, assembly of CLDN and OCLN proteins to TJs takes longer in the absence of ZO1 but does not block eventual establishment of the polarized epithelial structure with functional TJs within hours in cell culture [15]. However, KO of ZO1 and knockdown of ZO2 by RNA interference results in diffuse distribution of integral TJ proteins in epithelial cells with severe perturbation of the paracellular barrier [48]. While to our knowledge KO of 7H6 in epithelial cells has not yet been analyzed, its localization would suggest a paracellular barrier function [49,50]. In mice invivo KO or knockdown of TJ proteins results in a wide variety of phenotypes, ranging from a normal phenotype without any disease to lethality [51,52,53,54,55]. Furthermore, there are differences in the phenotype of TJ protein loss of function in mice and humans: e.g., while CLDN1 KO in a mouse model has shown to be lethal [52], congenital CLDN1 KO loss-of function mutations in human patients can manifest in a highly variable phenotype ranging normal health without disease to neonatal sclerosing cholangitis and ichthyosis of variable severity (NISCH syndrome), potentially due to compensatory upregulation of other CLDN members [56]. This indicates differential functions of the TJ orthologs in mice and humans and suggests that a complete loss of TJ proteins can be functionally compensated as shown for CLDN1 in humans.

### 2.2. Non-Junctional Localization of Tight Junction Proteins

Several TJ proteins have been described to be also localized outside of TJs at the basolateral membrane, in the cytoplasm, and in the nucleus. Non-junctional TJ proteins exert key regulatory functions on cell proliferation, cell adhesion, as well as migration and invasion [11]. As an example, CLDN1, 2, and 7 regulate cell-matrix interaction by forming complexes with integrin proteins at focal adhesions on the basolateral membrane of human lung, melanoma, colon, as well as breast cancer cells (Figure 1b) [57,58,59,60,61]. These interactions have not only been shown to affect epithelial adhesion to the matrix and cell proliferation [59], but also to be associated with cancer progression and metastasis [61]. The epithelial cell adhesion molecule (EpCAM) specifically stabilizes this non-junctional CLDN expression and regulates its lysosomal degradation (Figure 1b) [62]. In line with the potential pro-oncogenic function of CLDN proteins at the basolateral membrane, interaction of EpCAM with CLDN7 was reported to promote tumor progression and cell dissemination [63].

Several studies link basolateral CLDN expression with expression and activity of matrix metalloproteinases (MMPs) [64,65,66]. At the basolateral membrane of epithelial cells, secreted MMPs are able to degrade extracellular matrix proteins [67]. Interestingly, CLDN proteins have been shown to recruit and activate pro-MMP, hereby promoting migration and invasion of the corresponding cancer cells (Figure 1b) [68].

Nuclear localization has been reported for ZO1/ZO2 [69,70] and CLDN1-4 [71,72,73,74] in several types of cancer cells. The conditions or inducers under which these TJ proteins localize in the nucleus are poorly understood. However, in the case of CLDN1, phosphorylation by protein kinase A and C (PKA and PKC) has been shown to promote nuclear import [75]. Nuclear import of CLDN2 on the other hand is induced by dephosphorylation [72]. Functional investigations in colon cancer cells indicate nuclear localization of CLDN proteins to be associated with resistance to anoikis as well as migration and invasiveness [71], while nuclear localization of ZO1/ZO2 affects cell cycle progression and cell proliferation by transcriptional regulation of cyclin D1 in tumorous and non-tumorous epithelial cells [76,77] (Figure 1c).

## 3. Tight Junction Proteins and Their Role in Signaling

**Figure 2:**
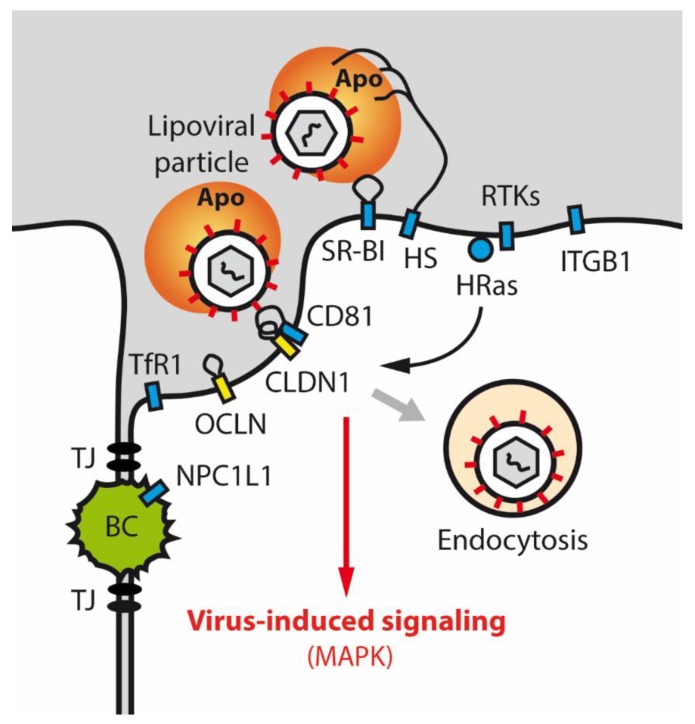
Hepatitis C virus (HCV) entry process and signaling. HCV lipoviral particle entry into hepatocytes requires a complex orchestration of entry factors that involves non-junctional TJ proteins CLDN1 and OCLN and virus-induced host signaling. Apo (Apolipoproteins), BC (Bile canaliculi), CD81 (Cluster of Differentiation 81), CLDN1 (Claudin-1), HRas (HRas Proto-Oncogene, GTPase), HS (Heparan sulfate), ITGB1 (Integrin Subunit Beta 1), MAPK (Mitogen-activated protein kinase), NPC1L1 (Niemann-Pick C1-like protein 1), OCLN (Occludin), RTK (Receptor tyrosine kinases), SR-BI (Scavenger Receptor Class B Member 1), TfR1 (Transferrin Receptor 1), TJ (Tight junction).

In colon and liver cancer cells, TJ proteins functionally crosstalk with key cellular signaling pathways, including PI3K/AKT, Wnt/β-catenin, and EGFR/ERK signaling [78,79,80]. Proteomic analysis of OCLN and CLDNs revealed numerous binding partners, that are known to be involved in cell signaling and trafficking, such as kinases, phosphatases, signaling adaptors, and receptor proteins [81,82]. A strong body of evidence indicates functional crosstalk of CLDN proteins with the EGFR signaling pathway. Dhawan et al. reported CLDN2 overexpression to promote cell proliferation in an EGFR-dependent manner in colon tumor cells [79]. De Souza et al. found EGF to increase CLDN3 expression via ERK and PI3K signaling, hereby accelerating colorectal tumor cell migration in vitro [83]. Finally, EGFR signaling has been shown to mediate the formation of a CD81-CLDN1 complex, hereby enabling entry of HCV into hepatocytes [82,84] (Figure 2).

Several studies further associate CLDN proteins with proapoptotic signaling. Singh et al. indicated CLDN1 as a driver of resistance to anoikis in colon cancer cells, a form of self-programmed death in epithelial cells following detachment from the surrounding extracellular matrix. Mechanistically, CLDN1 was found to directly interact with steroid receptor coactivator (Src), a non-receptor tyrosine kinase that binds to extracellular matrix proteins and plays a pivotal role in cellular signal transduction, promoting survival, proliferation, and angiogenesis in its activated form. The authors postulated the presence of a multiprotein complex consisting of CLDN1, ZO1, and Src2 that regulates activation of Src downstream oncogenic signaling [85]. Another cellular self-defense mechanism, Fas-mediated apoptosis, has been shown to alter OCLN and ZO1 expression in lung epithelia [86].

Furthermore, several studies indicate TJ proteins to function as intracellular signaling platforms, involved in regulation of cell differentiation and growth. Indeed, Spadaro et al. reported conformational changes of ZO1 to induce recruitment of the transcription factor DbpA to TJs in epithelial (Eph4) cells, hereby affecting cell proliferation [87]. In lung cells, interaction between CLDN18 and the signaling molecule Yes-associated protein (YAP) has been shown to affect colony formation and progenitor cell proliferation [88].

Posttranslational modification of TJ transmembrane proteins by growth factor signaling pathways fine-tune the TJ barrier function. Mitogen-activated protein kinase (MAPK) [89] and PKA [90] have been shown to phosphorylate CLDN1 at TJs of cerebral and lung endothelial cells, hereby affecting TJ permeability. Phosphorylation of CLDN5, induced by cyclic-AMP potentiates the blood–brain barrier [90], while PKA mediated phosphorylation of CLDN16 affects Mg2+ transport in kidney cells [91]. Vascular endothelial growth factor (VEGF) signaling perturbs hepatocellular TJ integrity by targeting OCLN via the PKC pathway [92]. Moreover, several studies indicate that cytokines, which are upregulated during inflammation, affect TJ protein expression. For example, Ni and coworkers demonstrated that TNF-α-induced phosphorylation of OCLN in human cerebral endothelial cells via MAPK, modulates TJ permeability [93]. Moreover, OCLN phosphorylation regulates its interaction with ZO1 in kidney cancer cells [94]. Exposure of intestinal epithelial cells with TNF-α hampers TJ permeability via NF-κB-dependent downregulation of ZO1 expression and altered junctional localization [95]. Loss of epithelial cell-to-cell junctions including TJs, represents a typical and early event in the evolution of epithelial-mesenchymal transition (EMT). EMT describes a process by which epithelial cells lose epithelial characteristics and acquire mesenchymal properties including the ability of migration and invasion [96,97].

## 4. Tight Junction Proteins in the Liver and the Blood-Biliary Barrier

Epithelial cells in the liver, namely hepatocytes and cholangiocytes, form the parenchymal structure of the organ and are characterized by a distinct cell polarity. TJs between neighboring hepatocytes separate the hepatocyte cell membrane into basal (sinusoidal), basolateral, and apical (bile canalicular) domains. By sealing the paracellular space, TJs and other adhesion complexes build the physiological BBIB, that segregates blood-containing basal hepatic sinusoids from apical bile canaliculi [9]. The BBIB hereby enables simultaneous execution of two major functions of the liver: the production and secretion of bile and the continuous metabolic exchange with the portal and systemic circulation allowing detoxification and excretion of proteins and coagulation factors. In particular, the apical bile canalicular domain of hepatocytes is characterized by numerous bile transporters and microvilli, that are required for bile secretion and absorption, while the basolateral sinusoidal domain is specialized in metabolic exchange with the blood [98]. CLDNs 1-3 and OCLN are expressed in TJs of hepatocytes and cholangiocytes [53,99,100]. While transmembrane TJ proteins on hepatocytes build the BBIB and shape bile canaliculi, TJs on cholangiocytes line the intrahepatic bile ducts [7]. The gallbladder on the other hand, shows physiologically strong expression of CLDNs 2, 3, 7, and OCLN. The hepatic sinusoidal endothelium strongly expresses CLDN5 [8].

In the normal liver and in contrast to other TJ proteins, tricellulin expression in hepatocytes and biliary epithelial cells strongly variates between individuals but is accentuated at tricellular contacts in colocalization with CLDN1 and CLDN4 [101]. In contrast to their weak expression on hepatocytes, the junctional adaptor proteins 7H6 and ZO1 are enriched in bile canaliculi [33,102]. 

KO studies in mice suggest a crucial role of CLDN2 and 3 for the BBIB. Thus, KO of the channel-forming CLDN2 lead to cholesterol gallstone disease due to a decrease in paracellular water transport [53]. CLDN3 KO in mice on the other hand, increases the paracellular phosphate ion transport of hepatic tight junctions, resulting in calcium phosphate core formation. Cholesterol overdose causes the cholesterol gallstone disease in these mice [99].

## 5. Tight Junction Proteins in Chronic Hepatobiliary Diseases

Chronic liver diseases constitute a global health problem, associated with high mortality due to its complications of liver cirrhosis and cancer [103]. Major causes comprise chronic hepatitis B virus (HBV) and HCV infection, alcoholic and metabolic liver disease such as non-alcoholic steatohepatitis. Decompensated liver cirrhosis is the fourth most common cause of death in adults in central Europe [104,105]. Downregulated expression or impaired function of TJ proteins have frequently been associated with chronic liver diseases [12]. Loss of the BBIB, which is maintained by junctional adhesion complexes including TJs represents a common feature in mice models of chronic liver injury [106,107]. Takaki et al. observed loss of TJ protein expression, including CLDN3 and ZO1 following hepatectomy and reappearance several days after surgery. This suggests a functional role of TJ proteins in liver regeneration [108]. Moreover, alterations related to the expression of TJ proteins have been implicated in chronic HCV infection, biliary diseases, and liver cancer. 

### 5.1. Tight Junction Proteins and HCV Infection

Chronic HCV infection represents a serious global health problem affecting more than 71 million people worldwide and potentially leads to liver fibrosis, cirrhosis, and hepatocellular carcinoma (HCC) [109,110,111]. Cell entry is a critical step in the HCV life cycle and involves a complex multi-step process consisting of viral attachment to the hepatocyte cell membrane and internalization [10,112]. HCV requires a complex orchestration of host dependency factors including among others CLDN1, OCLN, CD81, and SR-B1. Mechanistically, EGFR signaling promotes CLDN1-CD81 coreceptor association, which is a prerequisite for the internalization of the virus (Figure 2).

OCLN on the other hand, is believed to act downstream of the other cell entry factors CD81, CLDN1, and SRB1 during the HCV entry process [113,114]. OCLN interacts with HCV surface glycoprotein E2 via its extracellular loop 2 (ECL2) [115]. Of note, transgenic expression of human OCLN enables HCV infection of non-permissive species like mice [116,117,118]. However, the exact mechanism and localization of OCLN-HCV interaction is not fully understood. Considering its role for HCV cell entry, alterations in CLDN1 and OCLN expression levels and their functional consequences have been a focus of interest in the HCV field within the last years. Hepatic expression of CLDN1 and OCLN was found to be increased in liver biopsies of patients with chronic HCV infection [119]. In accordance, HCV liver graft infection is associated with OCLN and CLDN1 upregulation [120].

Anti-CLDN1 antibodies prevent and eliminate chronic HCV infection in cell-based and animal models without any detectable adverse effects and especially without disrupting TJ integrity or function [121,122,123,124]. The safety profile was further confirmed in human liver-chimeric mice and is most likely related to the molecular mechanism of action of CLDN1 monoclonal antibodies (mAbs) targeting the non-junctional expressed CLDN1 on hepatocytes without binding to CLDN1 localized in TJs [123,124,125]. Xiao et al. reported synergistic effects of anti-CLDN1 mAb with direct-acting antivirals as antiviral approaches for difficult-to-treat patients [126,127]. Confirming the functional role of OCLN in HCV entry, previous mechanistic monoclonal antibodies targeting ECL2 of OCLN were efficient in the prevention of infection both in cell culture and human liver chimeric mice without detectable side effects [114,128,129].

### 5.2. Tight Junction Proteins in Hepatocellular Carcinoma

Primary liver cancer is the sixth most frequent and second most deadly type of cancer in the world, with HCC being the most common histological subtype (75%–85%) [130]. Several members of the CLDN family have been reported to be perturbed during hepatocarcinogenesis. CLDN1, 4, 5, 7, and 10 are overexpressed in HCC [80,131,132,133,134,135]. Low levels of CLDN5 and high levels of CLDN7 were found to be independent prognostic factors [131]. Similarly, CLDN10 overexpression in HCC correlated with poor patients’ outcome and tumor recurrence [133,136]. In contrast, CLDN14 downregulation in HCCs correlates with advanced tumor stage and poor overall survival [137] and CLDN3 expression is decreased in HCC [138]. Bouchagier and coworkers reported an overexpression of OCLN in HCC tumors compared to non-neoplastic liver tissues, which positively correlated with a favorable prognosis [131]. Orban et al. on the other hand, found decreased OCLN mRNA and protein levels in HCC [102]. These opposing findings may be due to different histological grading of the analyzed HCC samples and a potential dedifferentiation characterized by decreased OCLN levels. Decreased cell migration and proliferation following treatment of HCC cells with different compounds was accompanied by upregulation of OCLN expression, indicating mesenchymal-epithelial transition (MET) [139,140,141] and thus supporting the findings from Bouchagier et al. Expression of tricellulin is very heterogeneous in HCC tissues, but seems to be positively correlated with poor prognosis [101]. Downregulation of ZO1 on the other hand, associates with poor prognosis in HCC patients undergoing hepatectomy [142]. Collectively, these studies suggest a pathogenic role of TJ proteins in hepatocarcinogenesis. 

Studies on TJ protein expression in chronic liver diseases together with clinical correlations are summarized in Table 1.

## 6. Tight Junction Proteins in Biliary Diseases

Considering that TJ proteins on bile canaliculi are major contributors to the BBIB, TJ integrity has frequently been investigated in biliary diseases. Indeed, disruption of bile duct epithelial barrier plays a crucial role in the pathogenesis of chronic biliary diseases [7]. Studies in animal models of cholestatic disease hereby revealed secondary expressional and morphologic alterations of the tight junctional network upon cholestatic liver injury [146]. Perturbation of TJ proteins could further be found in human biliary liver diseases as primary sclerosing cholangitis (PSC) [147] and cholangiocellular carcinoma (CCA) [148]. Moreover, primary perturbation of TJ proteins caused by homozygous mutations have been identified to account for cholestatic syndromes, including progressive familial intrahepatic cholestasis (PFIC) type 4 [149,150] and the neonatal ichthyosis-sclerosing cholangitis (NISCH) syndrome [151].

### 6.1. Tight Junction Proteins in Primary Biliary Cirrhosis and Secondary Sclerosing Cholangitis

Primary biliary cirrhosis (PBC) and PSC represent etiologies of chronic liver disease that are characterized by cholestasis and an increased risk of developing liver cirrhosis and cancer. Mediated by immunological mechanisms of bile duct destruction, patients typically present with elevated serum levels of bile acids [152,153]. Ultrastructural studies of damaged bile ducts in PBC show electron-dense deposits in enlarged intercellular spaces, infiltrated by immune cells indicating perturbated barrier integrity [154]. TJ proteins are responsible for the main barrier formations maintaining the BBIB and preventing bile regurgitation from the biliary tract. In this context, downregulation of the TJ proteins 7H6 and ZO1 in bile ducts in PBC and in hepatocytes in PSC has been suggested to account for the increased paracellular permeability observed in chronic cholestatic liver diseases. Consequently, toxic bile acids can enter the periductal area and promote the infiltration of immune cells, eventually leading to inflammatory driven progression of bile injury. Interestingly, the expression of these TJ proteins is preserved in PBC patients treated with ursodeoxycholic acid [147]. 

### 6.2. Primary Perturbation of Tight Junction Proteins in Biliary Diseases: NISCH Syndrome and PFIC Type 4

NISCH syndrome represents an extremely rare autosomal-recessive ichthyosis syndrome caused by mutations in the *CLDN1* gene leading to its abolished expression in liver and skin (KO phenotype). First being described in 2002, only 12 cases have been reported [151,155,156,157,158,159,160,161]. The clinical manifestation is variable ranging from absent or regressive cholestasis to progressive liver disease with liver failure. The hepatic feature of this syndrome is characterized by neonatal sclerosing cholangitis with elevated serum bile acids and hepatomegaly. Additional non-hepatic manifestations can include dental anomalies, mild psychomotor delay, ichthyosis, and scalp hypotrichosis as well as scarring alopecia [56,151]. The human phenotype hereby strongly deviates from the one observed in CLDN1-KO mice that present severely wrinkled appearance of the skin and death within 24 h after birth [52], indicating differential function of CLDNs in mice and humans. Thus, increased paracellular permeability and secondary bile injury due to CLDN1 absence in patients with NISCH syndrome [44] may be compensated by overexpression of other TJ protein members in the liver, explaining the variable phenotype [56]. Alternatively, mutations in other genes may be responsible for part of the observed phenotype. In conclusion, these findings demonstrate that CLDN1 is not essential for life in humans and its absence has a variable clinical phenotype. 

Loss of ZO2 on the other hand, is observed in PFIC type 4 [149,150]. Mechanistically, a mutation in the *ZO2* gene has been described to hamper proper localization of CLDN1 in TJs of cholangiocytes in the liver despite normal protein levels, hereby increasing paracellular permeability to bile acids [149]. Clinical signs of cholestasis appear within the first year of life in patients homozygous for this mutation and are typically contrasted by normal levels of γ-glutamyl transferase activity (GGT). Progressing into secondary biliary cirrhosis, affected patients present with severe liver disease at a young age, often requiring liver transplantation [149]. A missense mutation in the first PDZ domain of ZO2, that binds to CLDN1 in TJs has further been described in patients with familial hypercholanemia, characterized by pruritus and fat malabsorption but without progressive liver disease [162].

### 6.3. Tight Junction Proteins in Cholangiocellular Carcinoma

Cholangiocellular carcinoma (CCA) represents the second most common primary liver cancer type. With an overall incidence rate of 2/100000 it belongs to the rather rare cancer subtypes, though within the last few years, a dramatic increase in prevalence and mortality have been documented [163,164,165]. In contrast to the strong linkage of liver fibrosis/cirrhosis with HCC, most CCAs occur sporadically. However, known risk factors are PSC and HBV/HCV associated liver cirrhosis [166,167,168,169].

Several studies have reported evidence for potential functional implication of TJ proteins in CCA. CLDN3, 7, 8, and 10 expression were found to be decreased in intrahepatic CCAs compared to normal tissues. Significantly lower expression of CLDN1, 8, and 10 was also found in extrahepatic CCA, while CLDN1, 2, 3, 7, 8, and 10 are decreased in CCA of the gallbladder [148]. The most significant alteration of CLDN expression between CCA and adjacent liver tissue was found for CLDN10, as it was markedly decreased in all forms of bile duct cancers [148]. Moreover, in contrast to its restricted membrane localization in normal bile epithelia, intrahepatic CCA showed cytoplasmatic localization of CLDN10. Based on the negative staining in HCC and normal mature hepatocytes, CLDN4 and CLDN7 have been suggested as immunohistochemical markers of cholangiocellular differentiation in primary liver cancer [170,171]. In view of its preserved or even elevated expression in intra- and extrahepatic CCA, especially CLDN4 represents an attractive histological marker of CCA [148]. Interestingly, downregulation of CLDN4 by siRNA led to decreased migration and invasion of CCA cell lines [172]. CLDN18, that has been intensively studied in relation to gastric cancer is expressed in 40% of intrahepatic CCAs and is associated with lymph node metastasis and poor prognosis [173].

In intrahepatic CCA, tricellulin is decreased compared to adjacent tumor tissue, while patients with preserved tricellulin expression had significantly better clinical outcome and lower histological grading [101]. Downregulation of ZO1 and OCLN are associated with progression in biliary tract cancers [174]. 

All reported perturbations of TJ protein expressions in chronic hepatobiliary diseases are summarized in Table 2.

## 7. Summary

Tight junction proteins on hepatocytes and cholangiocytes play an important functional role as paracellular gatekeepers and represent the molecular basis of the BBIB, enabling exertion of two major function of the liver: production and secretion of bile as well as metabolic exchange and detoxification. Moreover, non-junctional TJ proteins at the basolateral membrane and in the nucleus exert key functions in cellular signaling, apoptosis, and migration. The TJ proteins CLDN1 and OCLN on the basolateral membrane of hepatocytes serve as entry factors for HCV—a major cause of liver disease and cancer worldwide. Highlighting its function as regulators of paracellular permeability enabling maintenance of the BBIB, secondary perturbation of TJ proteins has been described in biliary diseases, including PSC and PBC. In humans, the complete loss of distinct TJ proteins is not lethal, and the associated clinical phenotypes are highly variable as described for NISCH-syndrome or PFIC type 3. Finally, up- or downregulation of TJ protein expression in hepatobiliary cancer suggests a functional implication of TJ proteins in key cell regulatory signaling cascades potentially associated with carcinogenesis.

## Figures and Tables

**Table 1:** Perturbation of TJ proteins in chronic liver diseases.

Liver Disease	Tight Junction Protein	Perturbation	Potential Clinical Impact	References
HCV infection	CLDN1	Overexpression in chronically HCV- infected liver tissue Upregulation upon HCV liver graft infection	SNPs in *CLDN1* promoter confer susceptibility to HCV infection Crucial HCV entry factor, antiviral target	[143,144] [119,121,124], [120,122,123]
	OCLN	Overexpression in chronically HCV- infected liver tissue Upregulation upon HCV liver graft infection	Crucial HCV entry factor, antiviral target	[114,119,120,128,129,145]
HCC	CLDN1	Upregulated in the large majority of HCCs	Correlation of expression with patients’ survival Therapeutic target	[80,131,132,134,135]
	CLDN3, CLDN14	Downregulated/low expression in HCC	Unknown	[137,138]
	CLDN4, 5, 7 and 10	Upregulated in HCC	Unknown	[131] [133,136]
	OCLN	Both downregulated and upregulated described in HCC	Positive correlation of expression with good prognosis	[102,131]
	Tricellulin	Heterogeneous	Positive correlation with poor prognosis	[101]
	ZO1	-	Low expression correlates with HCC recurrence after hepatic resection	[142]

**Table 2:** Perturbation of TJ proteins in chronic biliary diseases.

Biliary Disease	TJ Protein	Perturbation	Potential Clinical Implication	References
Primary biliary cirrhosis (PBC)	ZO1	Downregulation in bile ducts of patients with PBC	Increased paracellular permeability Preservation of ZO-1 expression in patients treated with ursodeoxycholic acid	[147]
Primary sclerosing cholangitis (PSC)	ZO1	Downregulation on hepatocytes of patients with PSC	Increased paracellular permeability	[147]
Progressive familial intrahepatic cholestasis (PFIC) type 4	ZO2	Loss of expression	Failed localization of CLDN1 to TJs on cholangiocytes despite normal CLDN1 protein levels Increased paracellular permeability Progressive chronic liver disease	[149,150]
Familial hypercholanemia	ZO2	Missense mutation in the first PDZ domain of ZO2	Perturbed localization of CLDN1 in TJs Pruritus, fat malabsorption, elevated serum bile acid concentrations	[162]
NISCH syndrome	CLDN1	Loss of CLDN1 expression due to homozygous *CLDN1* mutation (functional KO)	Variable clinical outcome from mild to absent disease to neonatal sclerosing cholangitis and ichthyosis (with functional impact of additional mutations unknown) Increased paracellular permeability	[56,151]
CCA	CLDN1–3, 7, 8, and 10	Perturbed expression in intrahepatic, extrahepatic CCA, and/or CCA of the gallbladder	CLDN7: suggested as histological marker to distinguish CCA from HCC	[148,170]
	CLDN4	Perturbed expression in CCA	Suggested as histological marker to distinguish HCC and CCA	[148,170,171,172]
	CLDN18	Expressed in 40% of intrahepatic CCAs	Expression is associated with lymph node metastasis and poor prognosis	[173]
	Tricellulin	Downregulated in CCA	Positive correlation of expression with clinical outcome and low staging	[101]
	OCLN	Downregulated in CCA	Correlation of downregulated expression with tumor progression	[148]
	ZO1	Downregulated in CCA	Correlation of downregulated expression with tumor progression	[148]

## Abbreviations

Akt	AKT serine/threonine kinase
Apo	Apolipoprotein
BBIB	Blood-biliary barrier
bTJ	Bicellular tight junction
CCA	Cholangiocellular carcinoma
CD81	Cluster of differentiation 81
CLDN	Claudin
c-myc	MYC proto-oncogene
ECL2	Extracellular loop 2
EGFR	Epidermal growth factor receptor
EMT	Epithelial-mesenchymal transition
EpCAM	Epithelial cell adhesion molecule
ESAM	Endothelial cell-selective adhesion molecule
FAK	Focal adhesion kinase
GGT	γ-glutamyl transferase
HBV	Hepatitis B virus
HCC	Hepatocellular carcinoma
HCV	Hepatitis C virus
HRas	HRas proto-oncogene, GTPase
HS	Heparan sulfate
IgSF	Immunoglobulin superfamily
ILDR	Immunoglobulin-like domain containing receptor
ITGB1	Integrin subunit beta 1
JAM	Junctional adhesion molecules
KO	Knockout
LSR	Lipolysis-stimulated lipoprotein receptor
mAbs	Monoclonal antibodies
MAGI	Membrane-associated guanylate kinase inverted
MAPK	Mitogen-activated protein kinase
MARVEL	MAL and related proteins for vesicle trafficking and membrane link
MET	Mesenchymal-epithelial transition
MMP	Matrix metalloproteinase
NISCH	Neonatal ichthyosis-sclerosing cholangitis
NPC1L1	Niemann-Pick C1-like protein 1
OCLN	Occludin
PBC	Primary biliary cirrhosis
PFIC	Progressive familial intrahepatic cholestasis
PKA	Protein kinase A
PKC	Protein kinase C
PP	Protein phosphatase
PSC	Primary sclerosing cholangitis
RTK	Receptor tyrosine kinase
SH3	Src homology 3 domain
SNPs	Single nucleotide polymorphisms
SR-BI	Scavenger receptor class B member 1
Src	Steroid receptor coactivator
TfR1	Transferrin receptor 1
TJ	Tight junction
tTJ	Tricellular tight junction
TNF-α	Tumor necrosis factor alpha
VEGF	Vascular endothelial growth factor
YAP	Yes-associated protein
ZO	Zonula occludens
ZONAB	ZO1-associated nucleic acid binding protein
